# Supplementary material for: Novel 4D Tensor Decomposition-Based Approach Integrating Tri-Omics Profiling Data Can Identify Functionally Relevant Gene Clusters
Source: Biology (Basel). 2026 Jul 15;15(14):1155. doi: 10.3390/biology15141155 (PMC13405590; doi:10.3390/biology15141155)
Supplement: Supplementary file 1 [file biology-15-01155-s001.zip › biology-4358349-File S3.pdf]

Table S1: Factor Data Table of MOFA+

	mRNA	trans	protein
Factor1	7.53×10^{-2}	4.97×10^{1}	2.10×10^{-2}
Factor2	1.37×10^{1}	8.34×10^{0}	7.82×10^{-1}
Factor3	8.31×10^{0}	5.75×10^{-2}	5.19×10^{-2}
Factor4	4.01×10^{0}	3.16×10^{0}	1.78×10^{-1}
Factor5	3.06×10^{0}	1.87×10^{0}	2.03×10^{-1}
Factor6	1.70×10^{0}	9.21×10^{-1}	4.09×10^{-3}
Factor7	1.38×10^{0}	3.18×10^{-2}	2.20×10^{-3}
